# Mediation of exogenous hydrogen sulfide in recovery of ischemic post-conditioning-induced cardioprotection via down-regulating oxidative stress and up-regulating PI3K/Akt/GSK-3β pathway in isolated aging rat hearts

**DOI:** 10.1186/s13578-015-0003-4

**Published:** 2015-03-15

**Authors:** Hongzhu Li, Yuehong Wang, Can Wei, Shuzhi Bai, Yajun Zhao, Hongxia Li, Bo Wu, Rui Wang, Lingyun Wu, Changqing Xu

**Affiliations:** Department of Pathophysiology, Harbin Medical University, Baojian Road, Harbin, 150081 China; Department of Biology, Lakehead University, Thunder Bay, ON P7B5E1 Canada; Department of Health Science, Lakehead University, Thunder Bay, ON P7B5E1 Canada

**Keywords:** Hydrogen sulfide, Ischemic post-conditioning, Oxidative stress, Aging rats, Cardiomyocytes

## Abstract

The physiological and pathological roles of hydrogen sulfide (H_2_S) in the regulation of cardiovascular functions have been recognized. Cystathionine gamma-lyase (CSE) is a major H_2_S-producing enzyme in cardiovascular system. Ischemic post-conditioning (PC) provides cadioprotection in young hearts but lost in the aging hearts. The involvement of H_2_S in the recovery of PC-induced cardioprotection in the aging hearts is unclear. In the present study, we demonstrated that ischemia/reperfusion (I/R) decreased H_2_S production rate and CSE expression, aggravated cardiomyocytes damage, apoptosis and myocardial infarct size, reduced cardiac function, increased the levels of Bcl-2, caspase-3 and caspase-9 mRNA, enhanced oxidative stress in isolated young and aging rat hearts. I/R also increased the release of cytochrome *c* and down-regulated the phosphorylation of PI3K, Akt and GSK-3β in the aging rat hearts. We further found that PC increased H_2_S production rate and CSE expressions, and protected young hearts from I/R-induced cardiomyocytes damage, all of which were disappeared in the aging hearts. Supply of NaHS not only increased PC-induced cardioprotection in the young hearts, but also lightened I/R induced-myocardial damage and significantly recovered the cardioprotective role of PC against I/R induced myocardial damage in the aging hearts. LY294002 (a PI3K inhibitor) abolished but N-acetyl-cysteine (NAC, an inhibitor of reactive oxygen species, ROS) further enhanced the protective role of H_2_S against I/R induced myocardial damage in the aging hearts. In conclusion, these results demonstrate that exogenous H_2_S recovers PC-induced cardioprotection via inhibition of oxidative stress and up-regulation of PI3K-Akt-GSK-3β pathway in the aging rat hearts. These findings suggested that H_2_S might be a novel target for the treatment of aging cardiovascular diseases.

**Electronic supplementary material:**

The online version of this article (doi:10.1186/s13578-015-0003-4) contains supplementary material, which is available to authorized users.

## Introduction

After myocardium undergoes severe ischemia, restoration of blood flow is a prerequisite for myocardial salvage. However, reperfusion may also induce deleterious changes, such as decreased myocardial contraction and arrhythmias. These changes occur at the time of reperfusion, termed as “reperfusion injury” [1]. Cardiac damage in ischemic heart disease can be reduced by subjecting the heart to short bouts of ischemia before a prolonged ischemic episode, which process termed ‘ischemic preconditioning’ (IPC) [2]. However, IPC must be applied before the ischemic event, which in the clinical setting of acute myocardial infarction is unpredictable and impractical [3]. Zhao et al. reported that a similar regimen of brief periods of ischemia applied just after, instead of just before, sustained ischemia was as protective as preconditioning, named as ‘post-conditioning’ (PC) [4,5]. PC can be evoked by applying cycles of brief intermittent interruption of blood flow to the myocardium at the immediate onset of reperfusion after a prolonged period of ischemia. The fact that PC can be applied after a prolonged period of ischemia offers a novel approach to cardioprotection [3,6].

The involvement of several signaling elements, including radical oxygen species (ROS) [7,8], Ca^2+^ overload [9], prosurvival kinases such as phosphatidylinositol-3-kinase (PI3K)/Akt and its downstream component glycogen synthase kinase 3β (GSK3β) [10-12], extracellular signal-regulated kinase 1 and 2 (ERK 1/2) [13], mitochondrial ATP-sensitive potassium (mitoK_ATP_) channels [14], and mitochondrial permeability transition pore (mPTP) [13,15] in PC, has been investigated previously in separate studies. Some studies reported that the aged heart sustains increased injury during ischemia/reperfusion in both experimental models and in elderly patients [16-20]. Aging hearts are also resistant to the powerful endogenous protections provided by PC, in other words, PC lost myocardial protective effect in the aging hearts [20-22].

Hydrogen sulfide (H_2_S), a member of the gasotransmitter family, plays a number of important physiological roles within the body, including protection against cardiovascular diseases [23-26]. H_2_S is generated from the amino acid L-cysteine by three distinct enzymes: cystathionine gamma-lyase (CSE), cystathionine beta-synthase (CBS), and 3-mercaptopyruvate sulfurtransferase (MPST) [27,28]. In the cardiovascular system, CSE is the most abundantly expressed protein, and is responsible for the majority of endogenous H_2_S production [29]. As an important gasotransmitter in the cardiovascular system, H_2_S has important physiological functions, such as anti-atherosclerosis, anti-inflammatory, vasodilatation, protection of ischemia injury, and antioxidant effects, etc. [24,25,29-31]. Recent studies demonstrated that exogenous H_2_S postconditioning protected rat heart against ischemia and reperfusion injury through ATP-sensitive K^+^ (K_ATP_) channels opening and activation of several prosurvival kinases such as ERK1/2, PI3K/Akt, and protein kinase C (PKC) [32-37].

Several lines of evidence point to the implication of H_2_S signaling in the process of aging [38-40]. The level of cysteine is significantly decreased in the livers of older mice compared to young mice due to reduced activities of CSE and CBS [38]. The mRNA and protein levels of CSE are decreased in the lenses from old rats, and inhibition of CSE activity leads to cataractogenesis in vitro [41]. Recent study also indicated that H_2_S protected against cellular senescence via S-sulfhydration of Keap1 and activation of Nrf2 [42].

In the present study, we analyzed the effect of exogenous H_2_S on recovery of PC-induced cardioprotection and its possible mechanism, including oxidative stress and PI3K-Akt-GSK-3β pathway in the aging rats. Our study indicates an important role of H_2_S in protecting against the aging cardiovascular disease.

## Materials and methods

### Materials

Sodium hydrogen sulfide (NaHS), the anti-CSE antibody, LY294002 (a PI3K inhibitor) and N-acetyl-cysteine (NAC, an inhibitor of reactive oxygen species, ROS) were purchased from Sigma Chemical Co. (St. Louis, MO, USA). PI3K-Akt-GSK-3β antibodies were obtained from Cell Signaling Technology (Danvers, USA). The anti-cytochrome *c* (Cyt *c*) and GAPDH were from Santa Cruz (Bergheimer, Germany). The terminal deoxynucleotidyl transferase-mediated dUTP nick end labelling (TUNEL) kit was purchased from Roche (Mannheim, Germany). Assay kits for malondialdehyde (MDA), superoxide dismutase (SOD), lactate dehydrogenase (LDH), creatine kinase (CK) and ROS were purchased from Nanjing Jiancheng Bioengineering Institute (Nanjing, China). All other chemicals were from Sigma or Santa Cruz.

### Isolated heart preparation

The male Wistar young rats (3-month-old, 200–250 g) and the aging rats (24-months-old, 450–500 g) were used for this stud. All animal experiments were conducted in compliance with the Guide for the Care and Use of Laboratory Animals published by the China National Institutes of Health and approved by the Animal Care Committees of Harbin Medical University, China. The rats were anesthetized with 2% pentobarbital sodium (50 mg kg^−1^ intraperitoneally), and the hearts were mounted in a Langendorff perfusion apparatus and subjected to simulated ischemia/reperfusion (I/R) as described previously [5]. KH buffer comprised (in mmol/L): NaCl 118, KCl 4.7, MgSO_4_ 1.2, KH_2_PO_4_ 1.2, CaCl_2_ 2.5, NaHCO_3_ 25, and glucose 11 at pH 7.4. The KH was equilibrated with 95% O_2_ and 5% CO_2_ at 37°C for 20 min. The coronary flow rate was maintained at 12–13 ml/min during the stabilization, and with a constant pressure of 80 cm H_2_O throughout the experiment.

### Experimental protocols

**Figure 1:**
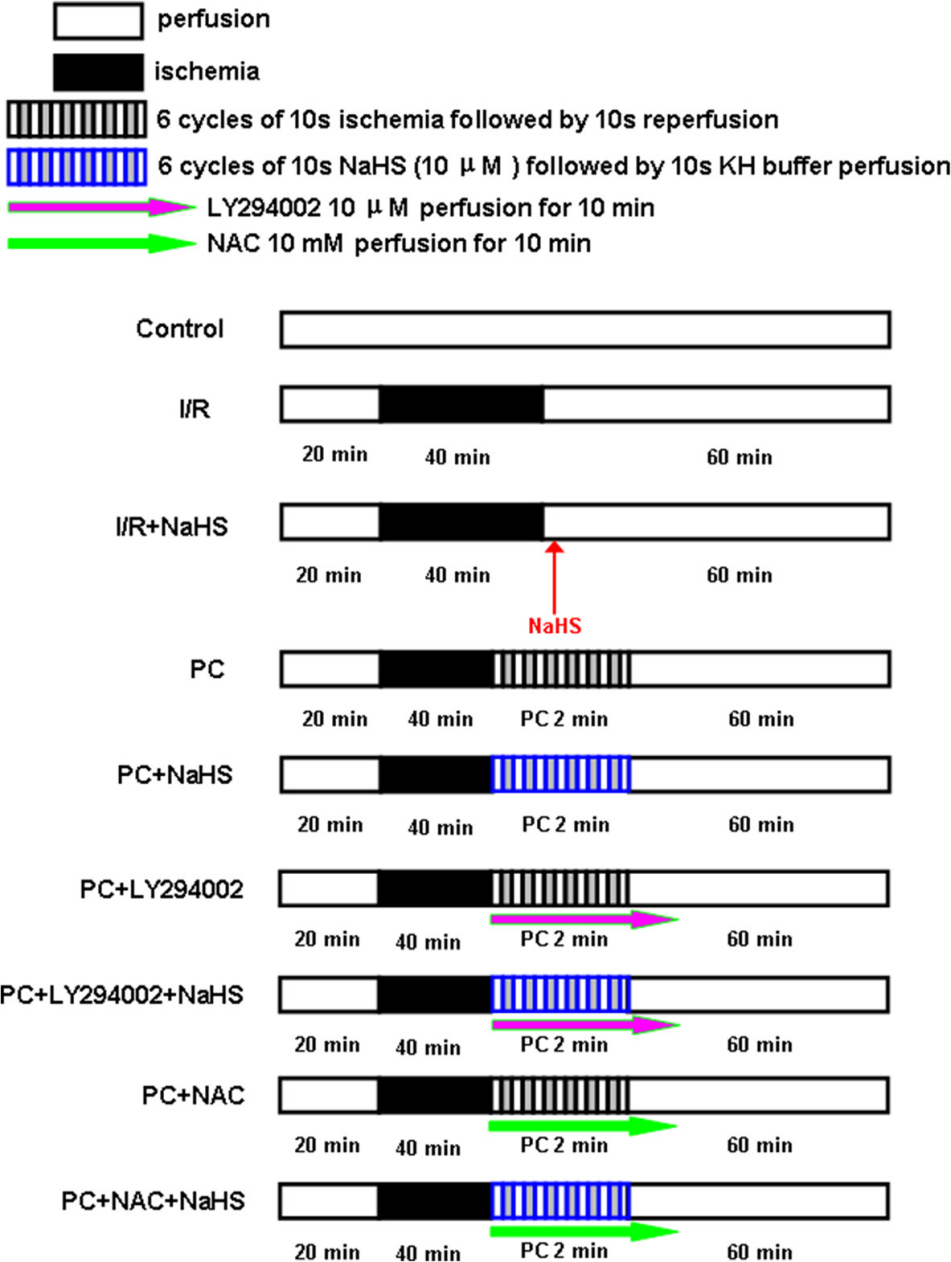
**Summary of experimental protocols.** After instrumentation and 20 min stabilization, all hearts subjected to 40 min global ischemia and 60 min reperfusion. For details of ischemic postconditioning, NaHS, LY294002 and NAC treatments see text.

Each heart was allowed to stabilize for 20 min. After the stabilization period, the hearts were subjected to a specific protocol: a 40 min of global no-flow ischemia by clamping the aortic cannula, followed by a period of 60 min reperfusion in all groups. Each group included 8 rats (n = 8) (Figure 1):

1. Control group: The isolated hearts (both young and aging rats) was perfused with standard KH buffer solution for 120 min.
2. I/R group: After stabilization, the hearts (both young and aging rats) were exposed to 40 min ischemia and then to 60 min reperfusion.
3. I/R + NaHS group: The procedure was similar to that for group 2, except that 10 μM NaHS were infused in 60 min reperfusion (both young and aging rats).
4. PC group: After stabilization, the hearts (both young and aging rats) were exposed to 40 min ischemia, and subjected to reperfusion for 10 s and to simulated ischemia solution for 10 s, repeated six times. They were then reperfused for 60 min.
5. PC + NaHS group: After stabilization, the hearts (both young and aging rats) were exposed to 40 min ischemia, initiated immediately at the onset of reperfusion, 10 μM NaHS were given at the onset of reperfusion for 10 s following with 10 s KH buffer. This protocol was repeated for another 5 times (total intervention time of 2 min). Other procedure was similar to that for group 3.
6. PC + LY294002 group: The procedure was similar to that for group 4, except that 10 μM LY294002 were injected into perfusate simultaneously via a side arm using a mechanical syringes pump for 10 min during early reperfusion (only aging rat hearts).
7. PC + LY294002 + NaHS group: The procedure was similar to that for group 5, except that 10 μM LY294002 were injected into perfusate simultaneously via a side arm for 10 min during early reperfusion (only aging rat hearts).
8. PC + NAC group: The procedure was similar to that for group 4, except that 10 mM NAC were injected into perfusate simultaneously via a side arm using a mechanical syringes pump for 10 min during early reperfusion (only aging rat hearts).
9. PC + NAC + NaHS group: The procedure was similar to that for group 5, except that 10 mM NAC were injected into perfusate simultaneously via a side arm for 10 min during early reperfusion (only aging rat hearts).

### Determination of H_2_S production

H_2_S production rate was measured as described previously [37]. In brief, after different treatments, the heart tissue was collected and homogenized in 50 mM ice-cold potassium phosphate buffer (pH 6.8). The flasks containing the reaction mixture (100 mM potassium phosphate buffer, 10 mM l-cysteine, 2 mM pyridoxal 5-phosphate, and 10% cell homogenates) and center wells containing 0.5 ml 1% zinc acetate and a piece of filter paper (2 × 2.5 cm) were flushed with N_2_ gas and incubated at 37°C for 90 min. The reaction was stopped by adding 0.5 ml of 50% trichloroacetic acid, and the flasks were incubated at 37°C for another 60 min. The contents of the center wells were transferred to test tubes, each containing 3.5 ml of water. Then 0.5 ml of 20 mM N, N-dimethyl-p-phenylenediamine sulfate in 7.2 M HCl and 0.5 ml 30 mM FeCl_3_ in 1.2 M HCl was added. The absorbance of the resulting solution at 670 nm was measured 20 min later with a FLUOstar OPTIMA microplate spectrophotometer.

### Measurement of MDA level, LDH, CK and SOD activities

At the end of 60 min of reperfusion, the coronary effluent liquid and the myocardial tissue were stored at −80°C until use. The myocardial tissue was homogenized in ice cold phosphate buffer to make a 10% homogenate. Then the homogenate was centrifuged at 3000 rpm for 15 min. Superoxide dismutase (SOD) and malondialdehyde (MDA) in the supernatant and lactate dehydrogenase (LDH) and creatine kinase (CK) the coronary effluent liquid were measured by using commercially available kits (Jiancheng Institute of Bioengineering, Nanjing, China). All assays were conducted according to the manufacturer’s instructions.

### Reactive oxygen species (ROS) level analysis

ROS level in the myocardial tissue was assayed as previously described [43,44] in which a non-polar compound dihydrodichlorofluorescein diacetate (H2 DCFH-DA), after conversion to a polar derivative by intracellular esterases, can rapidly react with ROS to form the highly fluorescent compound dichlorofluorescein. Briefly, the homogenate was diluted 1:20 times with ice-cold Locke’s buffer to obtain a concentration of 5 mg tissue/ml. The reaction mixture (1 ml) containing Locke’s buffer (pH 7.4), 0.2 ml homogenate or mitochondria (0.5 mg protein) and 10 ml of DCFH-DA (5 mM) was incubated for 15 min at room temperature to allow the DCFH-DA to be incorporated into any membrane-bound vesicles and the diacetate group cleaved by esterases. After 30 min of further incubation, the conversion of DCFH-DA to the fluorescent product DCF was measured using a spectrofluorimeter with excitation at 484 nm and emission at 530 nm. Background fluorescence (conversion of DCFH-DA in the absence of homogenate) was corrected by the inclusion of parallel blanks.

### Myocardial ultrastructure measurement

At the end of 60 min of reperfusion, heart tissue from left ventricular free wall (2–3 mm^3^) was immersed immediately in fixative (3.0% glutaraldehyde buffered in 0.1 M sodium cacodylate, pH 7.2). Following 2–3 days of storage, specimens were rinsed in PBS, postfixed in cacodylate-buffered 1% osmium tetroxide, dehydrated in an ethanol series, and embedded in polybed 812. Ultra-thin (90 nm) sections were made with microtome, and electron microscope images were digitally acquired and examined for signs of tissue damage.

### Observation of myocardial infarct size

At the end of 60 min reperfusion, all hearts were cut into five transverse slices parallel to the atrioventricular groove. Each slice was incubated for 15 min in a 1% solution of triphenyltetrazolium chloride (TTC) in phosphate buffer at 37°C. This method has been shown to reliably identify necrotic myocardium (which appears pale) from viable myocardium that stains brick red. The extent of the area of necrosis was quantified by computerized planimetry and corrected for the weight of the tissue slices. The total weight of the area of necrosis was calculated and expressed as a percentage of the total left ventricular weight [5].

### Cardiac function evaluation

A catheter was inserted into the left ventricle of the rat through the left atrium as described previously [5]. Briefly, the left ventricular end-diastolic pressure (LVEDP) was adjusted to 5–7 mmHg during initial equilibrium. The distal end of the catheter was connected to a Power Lab 8/SP TM data acquisition system (AD Instruments Incorporated, MA, Australia) via a pressure transducer for continuous recording of cardiac function. Cardiac function was evaluated based on left ventricular developed pressure (LVDP); LVEDP; and the positive and negative maximum rate of left ventricular pressure development (+dp/dt and -dp/dt).

### Apoptosis assay by TUNEL Staining

At the end of 60 min of reperfusion, the left ventricle free wall was collected and sliced into 3-mm^2^ size from each group. Paraffin-embedded, 4–5 μm-thick myocardial sections were used as described previously [5]. The apoptotic myocytes were stained by the TdT mediated dUTP nick end-labeling (TUNEL) assay using a Cell Death Detection Kit (Roche, Mannheim, Germany). Three sections from each myocardial sample were randomly selected, and ten microscopic fields per section were evaluated. The apoptotic index was determined by dividing the cell number of TUNEL-positive nuclei by the total number of cells and multiplying by 100.

### Real-Time PCR analysis

Total RNA was isolated using an RNeasy Mini Kit (Qiagen, Germantown, MD) and converted to cDNA with an iScriptTM cDNA Synthesis Kit (Bio-Rad, Hercules, CA). Real-time PCR was performed in an iCycler iQ5 apparatus (Bio-Rad) associated with the iCycler optical system software (version 3.1) using SYBR Green PCR Master Mix. The primers of Bcl-2 were 5′-GGCATCTTCTCCTTCCAG-3′ (forward) and 5′-CATCCCAGCCTCCGTTAT-3′ (reverse). Caspase-3 primers were 5′-CAGACAGTGGAACTGACGATGA-3′ (forward) and 5′-AACAGAAACATGCCCCTACCCC-3′ (reverse). Caspase-9 primers were 5′-CCCGTGAAGCAAGGATTT-3′ (forward) and 5′-ACTGTGGGTCTGGGAAGC-3′ (reverse). The primers for GAPDH were 5′-CTCAACTACATGGTCTACATG-3′ (forward) and 5′-TGGCATGGACTGTGGTCATGAG-3′ (reverse). The cycling conditions were: one cycle of 94°C for 2 min; 30 cycles of 94°C for 30 s, 60°C for 40 s and 72°C for 1 min; and 72°C for 4 min. Relative mRNA quantification was calculated by using the arithmetic formula “2-ΔΔCT”, where ΔCT is the difference between the threshold cycle of a given target cDNA and an endogenous reference GAPDH cDNA.

### Detection of Cyt c release from mitochondrial

Western blot analysis of Cyt *c* in the cytosolic fraction was performed as described previously [45]. Briefly, frozen heart samples were homogenized in ice-cold Tris-sucrose buffer (0.35 mM sucrose, 10 mM Tris–HCl at pH 7.5, 1 mM EDTA, 0.5 mM dithiothreitol, 0.1 mM phenylmethylsulphonyl fluoride). The homogenate was centrifuged at 1000 × g for 5 min at 4°C and the supernatant was further centrifuged at 40,000 × g for 30 min at 4°C. The supernatant was retained as the cytosolic fraction and analyzed by Western blot with a primary rat anti-Cyt *c* monoclonal antibody and a secondary goat anti-rat immunoglobulin G (Promage). GAPDH expression was used as the control.

### Western blotting analysis

Total proteins were prepared from cardiac tissue. Equal amounts of proteins were boiled and separated with SDS-PAGE and electrophoretically transferred to a nitrocellulose membrane, as described previously [45]. In each lane of a 10% sodium dodecyl sulfate-polyacrylamide gel electrophoresis, equal amounts of proteins were applied, electrophoresed and transferred to a polyvinylidene fluoride membrane. Membranes were blocked with Tris-buffered saline containing 5% non-fat milk at room temperature for 1h, then incubated overnight at 4°C with primary antibody. The primary antibody dilutions were 1:500 for CSE, 1:1000 for phosphorylated or total PI3K, Akt, GSK-3β and 1: 500 for Cyt *c* and GAPDH. The membrane was then washed three times with 1 × Tris-buffer saline-Tween 20 (TBST) buffer and incubated in TBST solution with horseradish peroxidase-conjugated secondary antibody (diluted 1:5,000) for 1 h at room temperature on a shaker. Finally, the membrane was washed with TBST solution for 3 times. The volume of the protein bands was quantified using a Bio-Rad Chemi Doc^TM^ EQ densitometer and Bio-Rad Quantity One software (Bio-Rad Laboratories, Hercules, USA).

### Statistical analysis

Statistical Analyses were performed with OriginPro 9.0 (OriginLab Corporation, MA) and SPSS 21.0 software (SPSS Inc, IL). All the data sets were tested for normality of distribution using the ShapiroWilks test and presented as either mean ± standard error of the mean or median range as appropriate. Comparison between 2 groups was performed by using the Student *t* test. Comparisons among 3 or more groups were performed by using 2-way analysis of variance (Tukey post hoc tests). The categorical data were analyzed with the Fisher exact test. Statistical significance level was set at p **<** 0.05.

## Results

### H_2_S production rate and CSE expressions in both young and aging hearts

**Figure 2:**
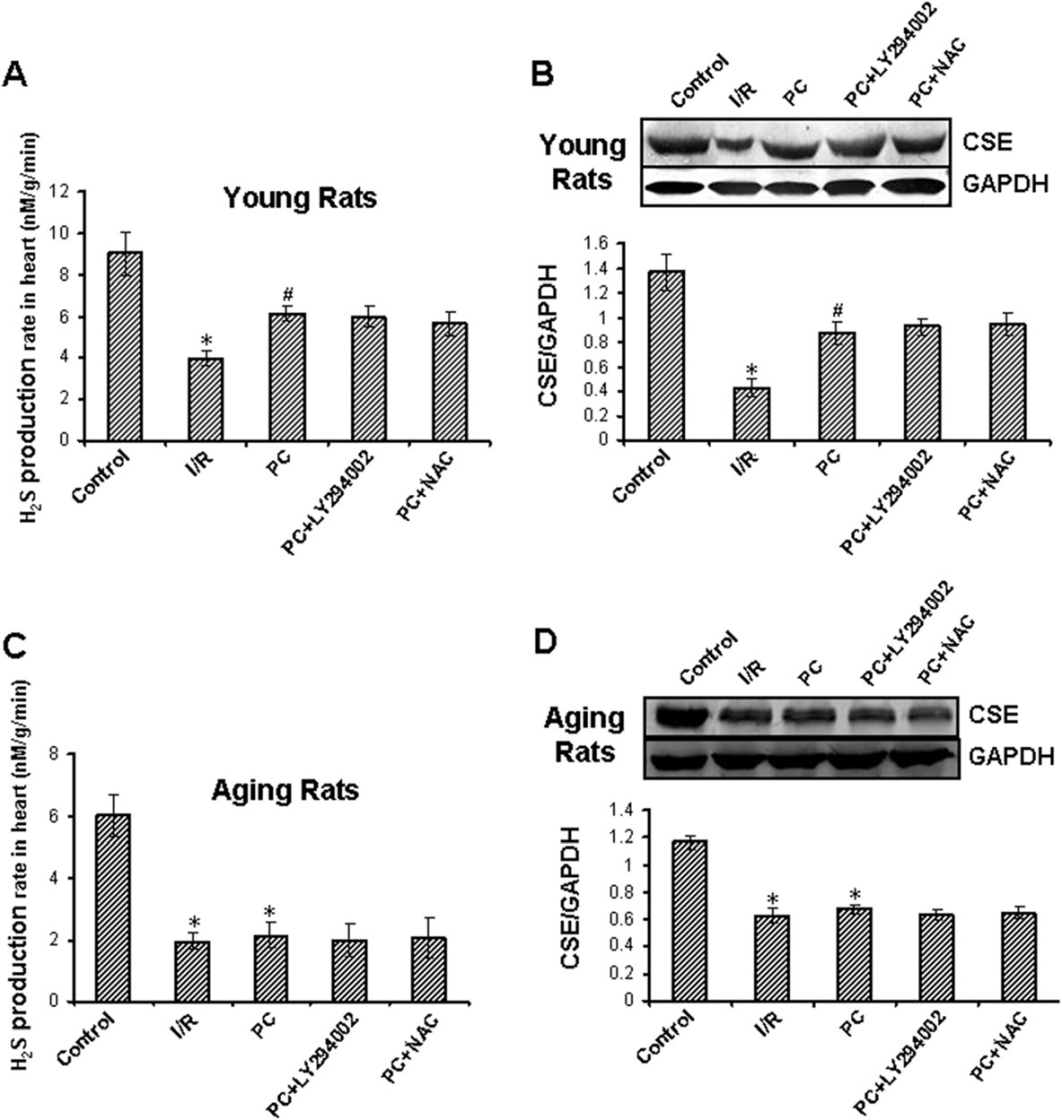
**H**_**2**_ **S production rate and CSE expressions.** The H_2_S production rate **(A and C)**. The expression of CSE protein **(B and D)**. The intensity of each band was quantified by densitometry, and data were normalized to the GAPDH signal. All data were from four independent experiments. * p < 0.05 *vs*. control group; # p < 0.05 *vs*. I/R group.

Compared with the control group, H_2_S production rate and CSE expressions were significantly decreased in the I/R group in both young and aging hearts (p < 0.05). PC increased H_2_S production rate and CSE expressions in the young hearts, but not in the aging hearts, in comparison with the I/R group. Compared with the PC group, LY294002 and NAC had no effect on H_2_S production rate and CSE expressions in both young and aging hearts (Figure 2A-D).

### Exogenous H_2_S attenuates myocardial tissue injury

In the young hearts, the activity of LDH and CK was incerased in the I/R group (p < 0.05 versus control group). Both NaHS (a H_2_S donor) and PC decreased the activity of LDH and CK (p < 0.05 versus I/R group). NaHS futher decreased the activity of LDH and CK (p < 0.05 versus PC group) (Additional file 1: Figure S1). Compared with the I/R group, both I/R + NaHS and PC decreased infarct size (p < 0.05), PC + NaHS futher decreased infarct size comparison with the PC group (p < 0.05) (Additional file 1: Figure S2).

**Figure 3:**
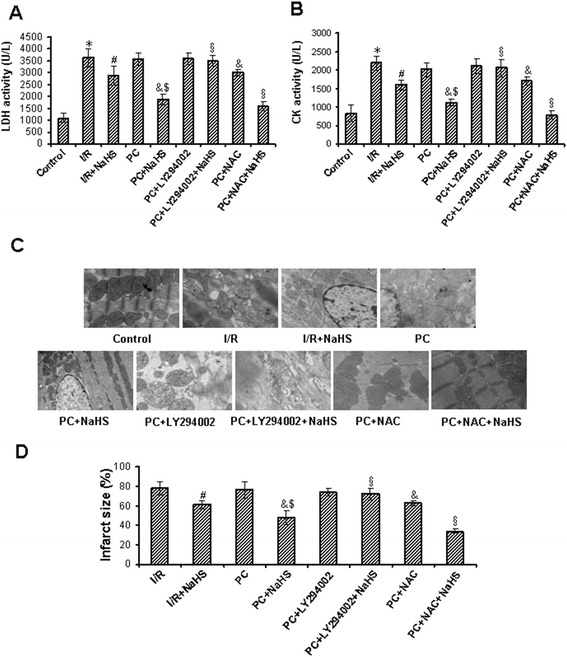
**The changes of myocardial tissue injury.** LDH **(A)** and CK **(B)** activities were detected in the coronary effluent liqud. Data are means ± S.E.M. of 8 determinations. * p < 0.05 *vs*. control group; # p < 0.05 *vs*. I/R group; & p < 0.05 *vs*. PC group; $ p < 0.05 *vs*. I/R + NaHS group. § p < 0.05 *vs*. PC + NaHS group. **(C)**. Ultrastructural changes in cardiomyocytes (magnification × 10000). Mitochondria structure was intact with well-organized myofilaments in the control group. Severe cell damage, including nuclear pycnosis, nuclear chromatin margination, aggregation and condensation, and swelling of mitochondria, and disruption of myofibrils were observed in the I/R and PC groups. Compared with I/R group, cardiomyocytes injury was lightened in the I/R + NaHS group.The cardiomyocytes injury was further lightened in the PC + NaHS group in comparison with the I/R + NaHS group. LY294002 canceled the effect of NaHS on the cardiomyocytes injury but NAC enhanced this effect of NaHS. **(D)**. A infarct size measured using TTC staining. Data were from four independent experiments. # p < 0.05 *vs*. I/R group; & p < 0.05 *vs*. PC group; $ p < 0.05 *vs*. I/R + NaHS group; § p < 0.05 *vs*. PC + NaHS group.

In the aging hearts, results of LDH and CK detections showed that compared with the control group, LDH and CK activities were significantly increased in the I/R and PC groups (p < 0.05), but the difference between I/R and PC groups was not significant. Compared with the I/R group, the activity of LDH and CK was decreased in the I/R + NaHS group (p < 0.05). The activity of LDH and CK was further decreased in the PC + NaHS group in comparison with the I/R + NaHS group (p < 0.05). LY294002 canceled the effect of NaHS on the activity of LDH and CK. In contract, NAC strengthened the effect of NaHS on the activity of LDH and CK (Figure 3A-B).

Morphological results showed that mitochondria structure was intact with well-organized myofilaments in the control group. Severe cell damage, including nuclear pycnosis, nuclear chromatin margination, aggregation and condensation, and swelling of mitochondria, and disruption of myofibrils were observed in the I/R and PC groups. Compared with I/R group, cardiomyocytes injury was lightened in the I/R + NaHS group. The cardiomyocytes injury was further lightened in the PC + NaHS group in comparison with the I/R + NaHS group. LY294002 canceled the effect of NaHS on the inhibiting cardiomyocytes injury. However, NAC increased this effect of NaHS (Figure 3C).

Infarct size was reduced in the I/R + NaHS group (p < 0.05 versus I/R group). Compared with I/R + NaHS group, infarct size was further reduced in the PC + NaHS group (p < 0.05). LY294002 abolished the beneficial effect of NaHS but NAC further enhanced the beneficial effect of NaHS (Figure 3D).

### Exogenous H_2_S improves cardiac function

In the young hearts, compared with the control group, cardiac function was aggravated (LVDP, +dp/dt and -dp/dt were decreased but LVDEP was increased) in the I/R group (p < 0.05). Both NaHS and PC improved cardiac function (p < 0.05 versus I/R group). PC + NaHS futher improved cardiac function (p < 0.05 versus PC group) (Additional file 1: Figure S3).

**Figure 4:**
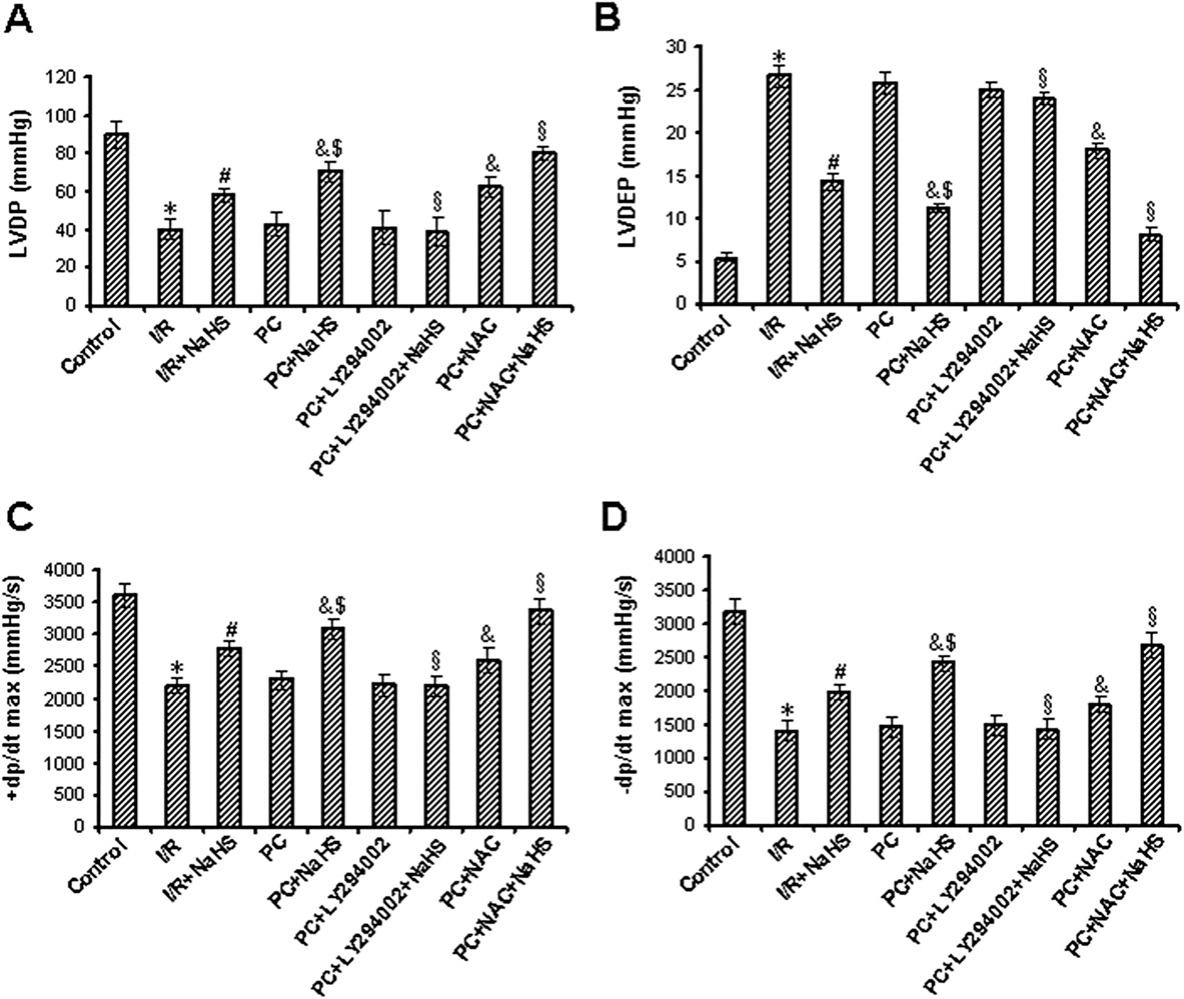
**Effect of various interventions on hemodynamic parameters in hearts. A**. LVDP, left ventricular developed pressure; **B**. LVEDP, left ventricular end-diastolic pressure; **C**. +dp/dt and **D**. -dp/dt, positive and negative maximum rate of left ventricular pressure development values are means ± S.E.M. of 8 determinations. * p < 0.05 *vs*. control group; # p < 0.05 *vs*. I/R group; & p < 0.05 *vs*. PC group; $ p < 0.05 *vs*. I/R + NaHS group; § p < 0.05 *vs*. PC + NaHS group.

In the aging hearts, compared with the control group, LVDP, +dp/dt and -dp/dt were decreased but LVDEP was increased in the I/R and PC groups (p < 0.05), and the difference between I/R and PC groups was not significant. Cardiac function was significantly improved in the I/R + NaHS group (p < 0.05 versus I/R group), it was further improved in the PC + NaHS group (p < 0.05 versus I/R + NaHS group). LY294002 abolished the beneficial effect of NaHS on the cardiac function but NAC increased this effect of NaHS (Figure 4A-D).

### Exogenous H_2_S decreases the rate of apoptotic cell

In the young hearts, compared with the control group, the rate of apoptotic cell was increased in the I/R group (p < 0.05). Compared with the I/R group, the rate of apoptotic cell was decreased in the I/R + NaHS and PC groups (p < 0.05). The rate of apoptotic cell was futher decreased in the PC + NaHS group (p < 0.05 versus PC group) (Additional file 1: Figure S4A).

**Figure 5:**
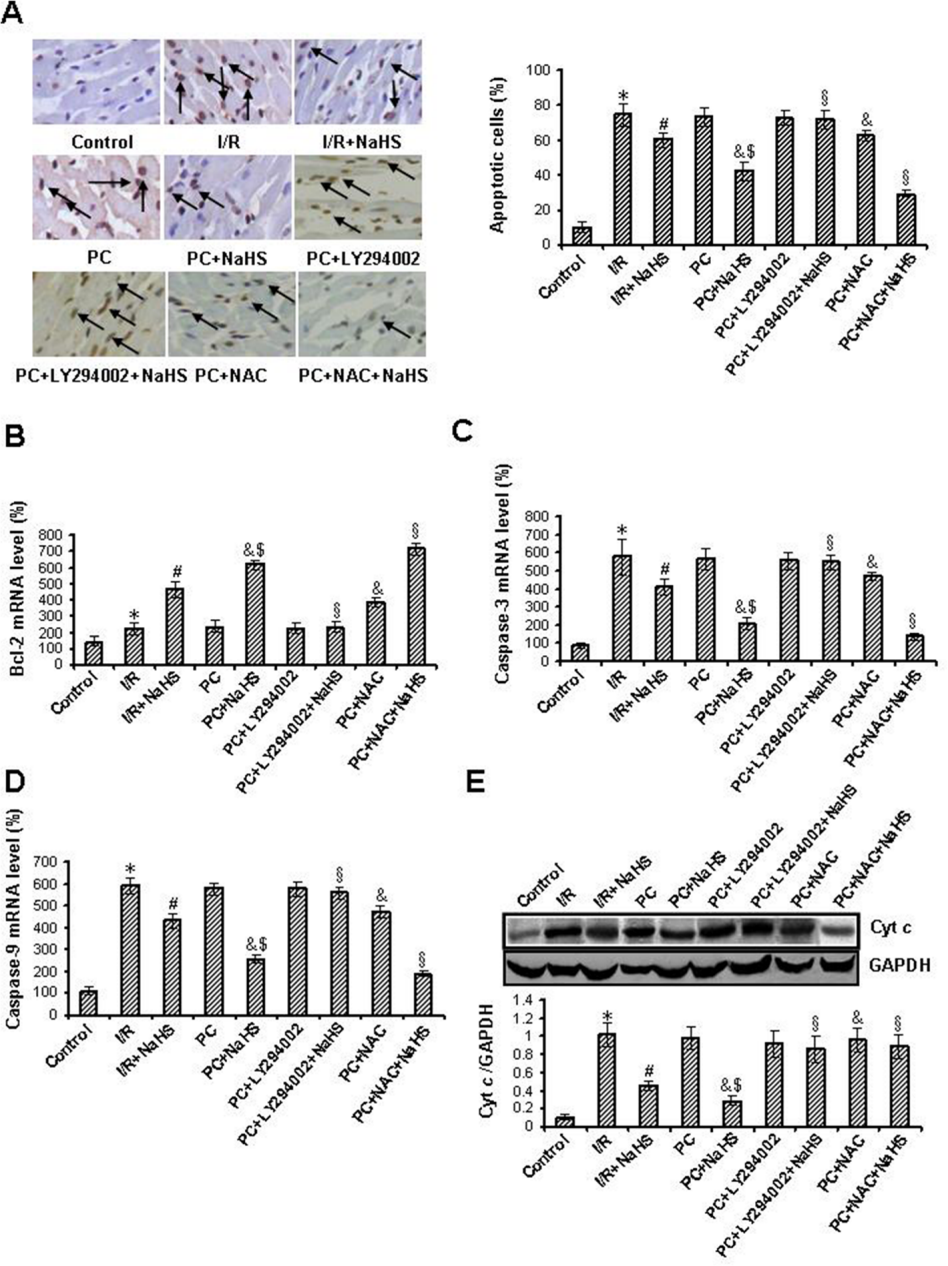
**The changes of cardiomyocytes apoptosis and related factors with apoptosis. (A)**. TUNEL staining detected cardiomyocytes apoptosis. Nuclei with brown staining indicate TUNEL positive cells (400×). The cells of arrow are apoptotic cells. The level of Bcl-2 **(B)**, caspase-3 **(C)** and caspase-9 mRNA **(D)**. The level of Bcl-2, caspase-3 and caspase-9 mRNA was tested using Real-Time PCR. The data were normalized to the GAPDH. **(E)**. Release of Cyt *c*. The intensity of each band was quantified by densitometry, and data were normalized to the GAPDH signal. All data were from four independent experiments. * p < 0.05 *vs*. control group; # p < 0.05 *vs*. I/R group; & p < 0.05 *vs*. PC group; $ p < 0.05 *vs*. I/R + NaHS group; § p < 0.05 *vs*. PC + NaHS group.

In the aging hearts, only 8 ± 2.9% TUNEL positive nuclei were detected in control group, and I/R and PC significantly increased the percentage of apoptotic cells to 74 ± 6.3% and 71 ± 8.5%, respectively (p < 0.05 versus control group). Compared with the I/R group, the percentage of TUNEL-positive cells was to 60 ± 4.0% in the I/R + NaHS group (p < 0.05). The percentage of TUNEL-positive cells in the PC + NaHS group was further decreased (42 ± 6.0%) compared with I/R + NaHS group (p < 0.05). LY294002 canceled the effect of NaHS on the apoptosis but NAC enhanced this effect of NaHS (Figure 5A).

### Exogenous H_2_S increases the level of Bcl-2 mRNA, decreases the level of caspase-3 and caspase-9 mRNA and Cyt *c* release

In the young hearts, the level of Bcl-2, caspases-3 and caspase-9 mRNA was increased in the the I/R group in comparison with the control group (p < 0.05). Compared with the I/R group, Bcl-2 mRNA levels were increased, caspases-3 and caspase-9 mRNA levels were decreased in the I/R + NaHS and PC groups (p < 0.05). the level of Bcl-2 was futher increased, the level of caspases-3 and caspase-9 mRNA was further decreased the PC + NaHS group in comparison with the PC group (p < 0.05) (Additional file 1: Figure S4B).

In the aging hearts, compared with the control group, the level of Bcl-2, caspases-3 and caspase-9 mRNA and the mitochondrial release of Cyt *c* were significantly increased in the I/R and PC groups (p < 0.05), but the difference between I/R and PC groups was not significant. The level of caspases-3 and caspase-9 and the release of Cyt *c* were obviously decreased, but Bcl-2 mRNA levels were increased in the I/R + NaHS group (p < 0.05 versus I/R group). Compared with the I/R + NaHS group, caspases-3 and caspase-9 mRNA levels and the release of Cyt *c* were further decreased, but Bcl-2 mRNA levels were further increased in the PC + NaHS group (p < 0.05). LY294002 canceled the effect of NaHS on the level of Bcl-2, caspases-3 and caspase-9 mRNA and the release of Cyt *c* but NAC increased this effect of NaHS (Figure 5B-E).

### Exogenous H_2_S inhibits SOD activity and decreases MDA content and ROS level

In the young hearts, compared with the control group, I/R decreased SOD level, increased MDA and ROS levels (p < 0.05). SOD level was increased, MDA and ROS levels were decreased in the I/R + NaHS and PC groups (p < 0.05 versus I/R group), the level of SOD was further increase, the level of MDA and ROS was further decreased in the PC + NaHS group in comparison with the PC group (p < 0.05) (Additional file 1: Figure S5).

**Figure 6:**
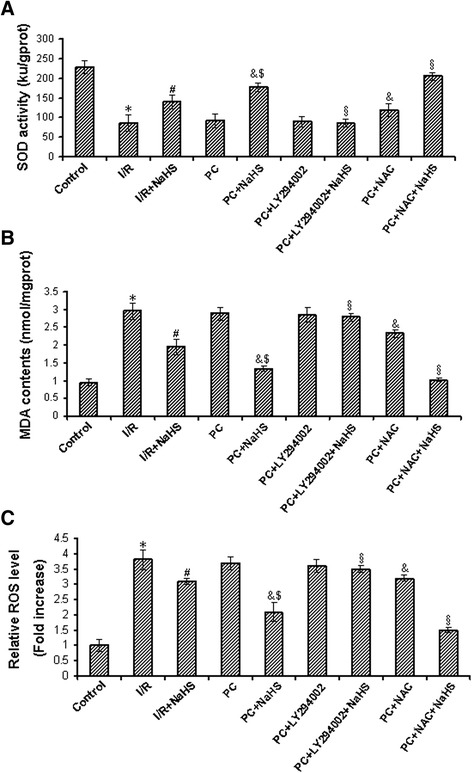
**The changes of oxidative stress related factors.** SOD **(A)** activity, MDA **(B)** content and ROS **(C)** level. All data were from eight independent experiments. * p < 0.05 *vs*. control group; # p < 0.05 *vs*. I/R group; & p < 0.05 *vs*. PC group; $ p < 0.05 *vs*. I/R + NaHS group; § p < 0.05 *vs*. PC + NaHS group.

In the aging hearts, SOD activity was reduced, MDA content and ROS level were increased in the I/R and PC groups (p < 0.05 versus control group), but the difference between I/R and PC groups was not significant. Compared with the I/R group, the activity of SOD was increased, the content of MDA and the level of ROS were decreased in the I/R + NaHS group (p < 0.05). PC + NaHS further increased SOD activity and decreased MDA content and ROS level (p < 0.05 versus I/R + NaHS group). LY294002 abolished the effect of NaHS on the oxidative stress but NAC increased this effect of NaHS (Figure 6A-C).

### Exogenous H_2_S up-regulates PI3K–Akt–GSK-3β pathway

**Figure 7:**
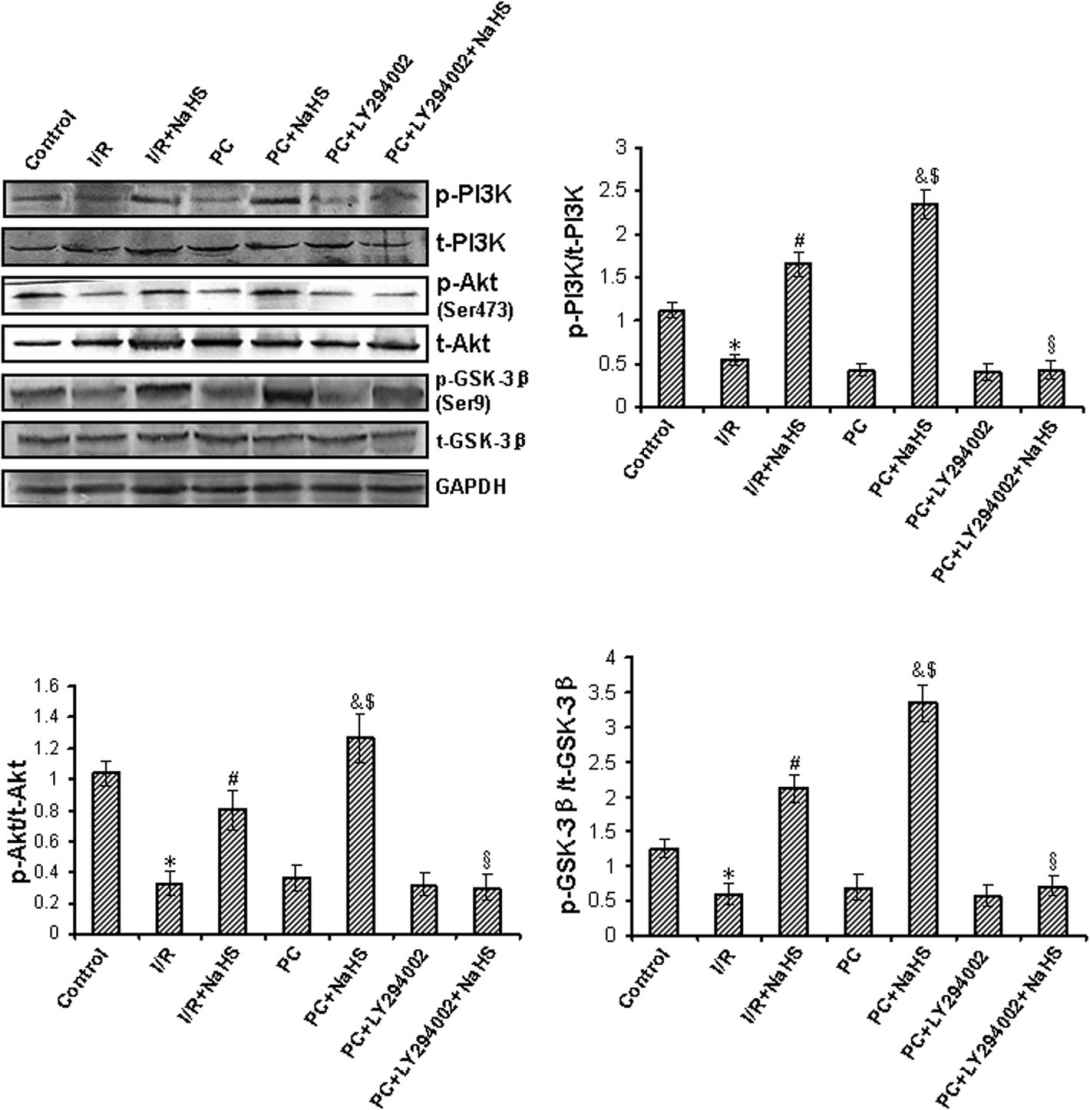
**The change of PI3K-Akt-GSK-3β pathway.** The phosphorylation of PI3K, Akt and GSK-3β was detected using Western bloting. The graphs represent the optical density of the bands of phosphorylated PI3K, Akt and GSK-3β normalized with the expression of total PI3K, Akt and GSK-3β, respectively. All data were from four independent experiments. * p < 0.05 *vs*. control group; # p < 0.05 *vs*. I/R group; & p < 0.05 *vs*. PC group; $ p < 0.05 *vs*. I/R + NaHS group; § p < 0.05 *vs*. PC + NaHS group.

In the aging hearts, our results showed that the levels of phosphorylated PI3K, Akt, GSK-3β were decreased in the I/R and PC groups compared with that in control group (p < 0.05), but the difference between I/R and PC groups was not significant. Phosphorylation of PI3K, Akt, GSK-3β was increased in the I/R + NaHS group compared with that in I/R group (p < 0.05). PC + NaHS further increased the levels of phosphorylated PI3K, Akt, GSK-3β (p < 0.05 versus I/R + NaHS group) (Figure 7). The total amount of PI3K, Akt, GSK-3β protein remained unchanged with different stimulations (Figure 7). We further found that LY294002 significantly suppressed H_2_S induced phosphorylation of PI3K, Akt, GSK-3β (Figure 7).

## Discussion

I/R injury promotes production of ROS, abnormal lipid metabolism, calcium overload and apoptosis etc. [1]. The mechanisms of apoptosis include activation of mitochondrial, death receptors and endoplasmic reticulum stress pathways, the most important of which is the mitochondrial pathway [1,46]. Cyt *c* is the initiating factor of mitochondrial apoptosis pathway. The Cyt *c* is released from injured mitochondria and triggers cytosolic caspase-3 activation through formation of the cytochrome c/Apaf-1/caspase-9- containing complex apoptosome and then lead to apoptosis [1,46]. Bcl-2 belong to a potent inhibitor of apoptosis and inhibit the mitochondria disruption and the subsequent Cyt *c* release, and the activation of caspase [1,46].

PC was firstly described by Zhao et al. to reduce myocardial injury to an extent comparable to ischemic preconditioning, offering a novel approach to myocardial protection [4,33]. PC prevents myocardial I/R injury by decreasing the leakage of myocardial enzymes (LDH, CK etc.), lowering apoptosis and infarct size, improving cardiac function and inhibiting oxidative stress etc. PC plays cadioprptection in the adult rats, but not in the aging rats [21]. Aging affects cardiomyocytes at several subcellular and molecular levels, including alterations in the gene/protein expression and posttranslational modifications such as advanced glycation endproducts and protein oxidation, changes at the DNA level for example mutations and telomere shortening, oxidative stress increase (ROS formation), and decrease of autophagy [21]. Taken together, cardiomyocytes undergo complex changes, which finally result in loss of contractile function and endogenous protection against irreversible injury in the aging rats. So PC lost myocardial protective effect in the aging rats. Our previous results in both the I/R model of the young rats and hypoxia/reoxygenation (H/R) model of primary cultural neonatal cardiomyocytes demonstrated that PC attenuated I/R or H/R induced-cardiomyocytes injury and apoptosis [1,5,45]. In present study, we also found that PC inhibited I/R induced-myocardial tissue injury and apoptosis in the young hearts (Additional file 1: Figure S1–S5). The I/R aggravated cardiomyocytes damage, apoptosis and myocardial infarct size, reduced cardiac function, increased the level of Bcl-2, caspases-3 and caspase-9 mRNA, the mitochondrial release of Cyt *c* and oxidative stress in the aging hearts. PC did not change the above mentioned indexes in comparison with the I/R (Figures 3, 4, 5 and 6). Taken together, our results indicated that aging rat hearts suffered from I/R injury and PC did not improve this injury. How to restore PC to aging myocardial protective effects has become the focus.

As well know, H_2_S is as the third endogenously produced gaseous signaling molecule, in addition to nitric oxide and carbon monoxide [29]. H_2_S produced by CSE in cardiovascular system acted as a physiological cardiac function regulator, which protected the cardiac function in ischemic reperfusion or hypoxia [33,47]. In the present study, we found that I/R reduced H_2_S production rate and CSE expression in both young and aging hearts, while PC only up-regulated CSE/H_2_S system in the young rats but not in aging hearts (Figure 2). We thought the loss of PC cardioprotection in the aging rats may be related with the decrease of endogenous H_2_S. Therefore, we used NaHS solution as a source of H_2_S and investigated that NaHS lightened I/R injury and improved ptotection of PC on I/R injury via the decreasing cardiomyocyte damages, improving cardiac function, inhibiting apoptosis and oxidative stress in the aging rats. These suggest that exogenous H_2_S recovered PC-induced cardioprotective effects in the aging rats.

ROS of physiological conditions are generated at low levels and play major roles in signaling and metabolic pathways [48,49], however, under pathologic conditions for example I/R, their overproduction lead to oxidative stress, causing cell damage such as DNA oxidation, chain reactions of membrane lipid peroxidation, and alterations of membrane fluidity [48,50,51]. ROS produces MDA, an end product of lipid peroxidation. The level of MDA reflects the extent of ROS. The overproduction of ROS can be detoxified by endogenous antioxidants, causing their cellular stores to be depleted [52]. SOD is thought to be one dominant enzymes acting as free radical scavengers that could prevent the deleterious stroke-induced ROS generation [53]. SOD scavenges the superoxide anion radical by catalyzing its dismutation to H_2_O_2_ [51]. Our result showed that the ROS level and MDA content were markedly decreased and SOD activity was increased in the PC + NaHS group compared with both PC and I/R + NaHS groups. Meanwhile, LY294002 (an inhibitor of PI3K) abolished the effect of NaHS on the oxidative stress and NAC (an inhibitor of ROS) increased this effect of NaHS (Figure 6). These data suggest that exogenous H_2_S recovered PC-induced cardioprotection through the inhibition of oxidative stress in the aging rats.

A lot of signaling pathways are involved in PC-induced cardioprotection [10-13]. PI3K-Akt-GSK-3β pathway is one of the most important pathways [10]. PI3K can phosphorylate Akt, an initiator of the downstream pathways in inhibiting apoptosis [54]. It phosphorylates Bad and ultimately inhibits Cyt *c* release by blocking the channel formed by Bcl-2-associated X protein (Bax) in the mitochondrial membrane [54]. Moreover, Akt can phosphorylate GSK3β to prevent mPTP opening [54]. We found that, compared with both PC and I/R + NaHS, PC + NaHS increased phosphorylated PI3K, Akt and GSK-3β. LY294002 canceled the beneficial effect of NaHS (Figure 7). The present study demonstrates that exogenous H_2_S recovered PC-induced cardioprotective effects by up-regulating PI3K-Akt-GSK-3β pathway in the aging rats.

**Figure 8:**
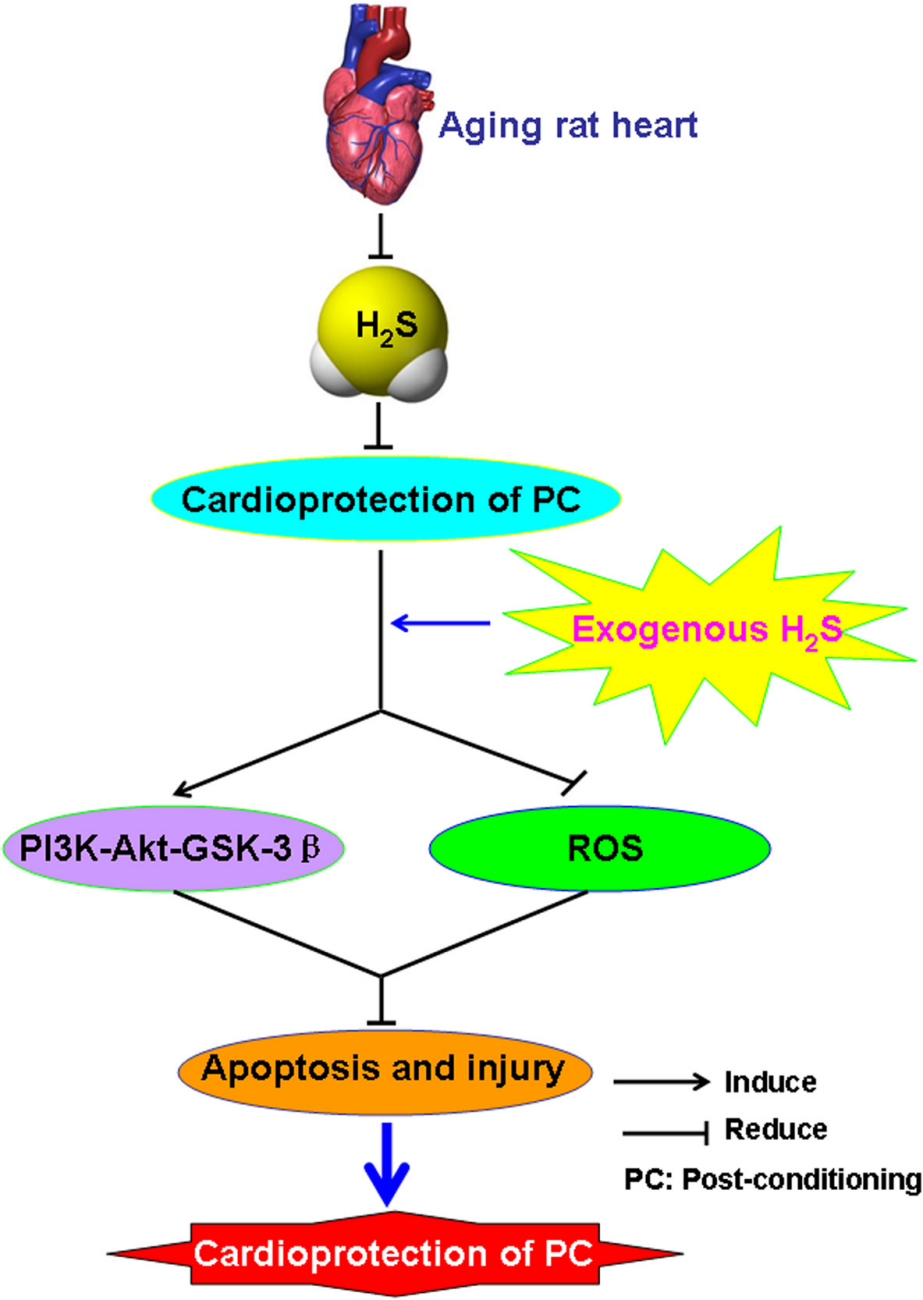
**Exogenous hydrogen sulfide involved in recovery of PC-induced cardioprotection in isolated aging rat hearts.** Exogenous H_2_S recovers PC-induced cardioprotection by up-regulating PI3K-Akt-GSK-3β pathway and inhibiting oxidative stress in the aging rats.

In summary, this study demonstrated for the first time that H_2_S plays an important role in the recovery of PC-induced cardioprotection in the aging rats. This effect is mediated via up-regulating PI3K-Akt-GSK-3β pathway and preventing oxidative stress (Figure 8).

## Additional file

Additional file 1: Figure S1. The effect of exogenous H_2_S on the activity of LDH and CK in the young rat hearts. **Figure S2.** The effect of exogenous H_2_S on infarct size in the young rat hearts. **Figure S3.** The effect of exogenous H_2_S on cardiac function in the young rat hearts. **Figure S4.** The effect of exogenous H_2_S on apoptosis in the young rat hearts. **Figure S5.** The effect of exogenous H_2_S on the level of SOD, MDA and ROS in the young rat hearts.
