# Supplementary material for: Mediation of exogenous hydrogen sulfide in recovery of ischemic post-conditioning-induced cardioprotection via down-regulating oxidative stress and up-regulating PI3K/Akt/GSK-3β pathway in isolated aging rat hearts
Source: Cell Biosci. 2015 Mar 15;5:11. doi: 10.1186/s13578-015-0003-4 (PMC4364662; doi:10.1186/s13578-015-0003-4)
Supplement: Additional file 1: Figure S1. — The effect of exogenous H2S on the activity of LDH and CK in the young rat hearts. Figure S2. The effect of exogenous H2S on infarct size in the young rat hearts. Figure S3. The effect of exogenous H2S on cardiac function in the young rat hearts. Figure S4. The effect of exogenous H2S on apoptosis in the young rat hearts. Figure S5. The effect of exogenous H2S on the level of SOD, MDA and ROS in the young rat hearts. [file 13578_2015_3_MOESM1_ESM.doc]

**Supplemental Data Files for**

**Mediation of exogenous hydrogen sulfide in recovery of ischemic post-conditioning-induced cardioprotection via down-regulating oxidative stress and up-regulating PI3K/Akt/GSK-3β pathway in isolated aging rat hearts**

Hongzhu Li, Yuehong Wang, Can Wei, Shuzhi Bai, Yajun Zhao, Hongxia Li,

Bo Wu, Rui Wang, Lingyun Wu, Changqing Xu

**Supplemental Results**

**
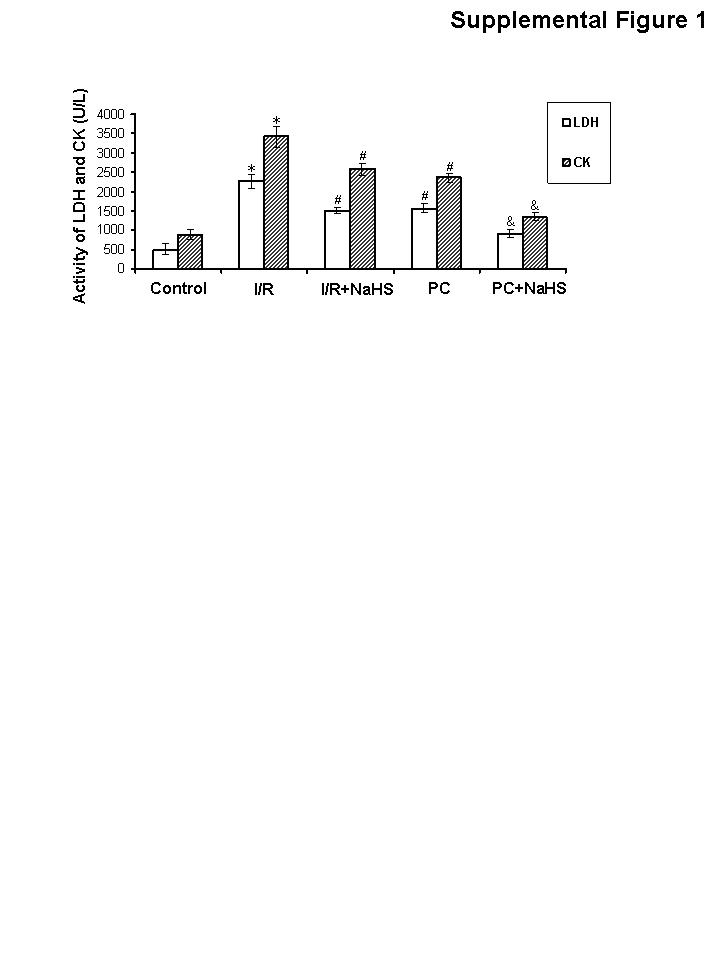
**

**Figure 1. The effect of exogenous H_2_S on the activity of LDH and CK in the young rat hearts.** LDH and CK activities were detected in the coronary effluent liquid. Data are means ± S.E.M. of 8 determinations. * p<0.05 *vs*. control group; # p<0.05 *vs*. I/R group; & p<0.05 *vs*. PC group.


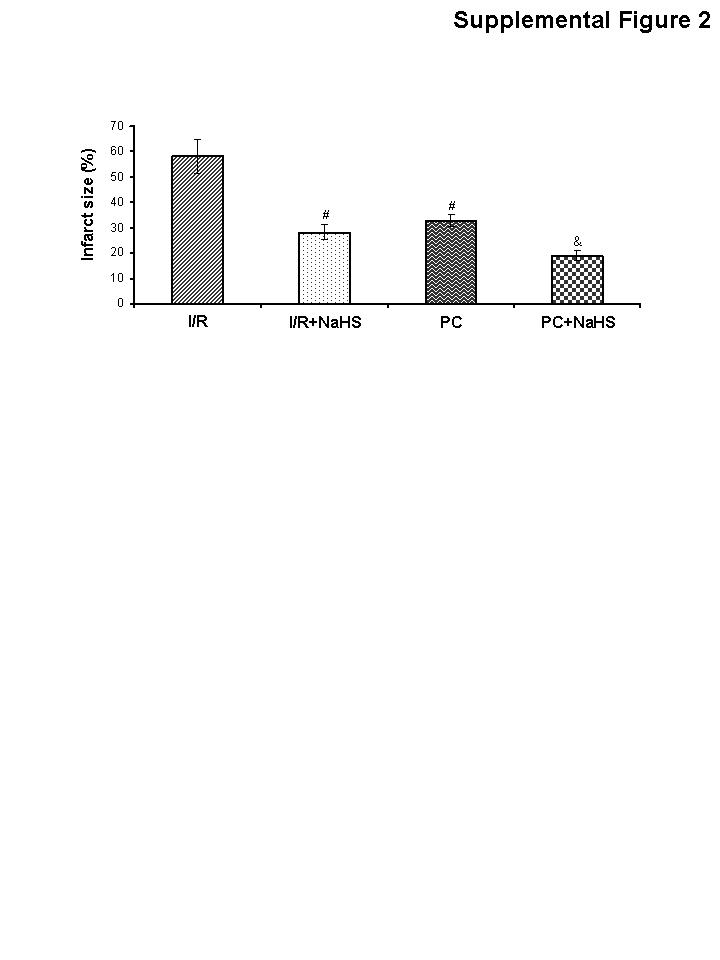


**Figure 2. The effect of exogenous H_2_S on infarct size in the young rat hearts.**. A infarct size measured using TTC staining. Data were from four independent experiments. # p<0.05 *vs*. I/R group; & p<0.05 *vs*. PC group.


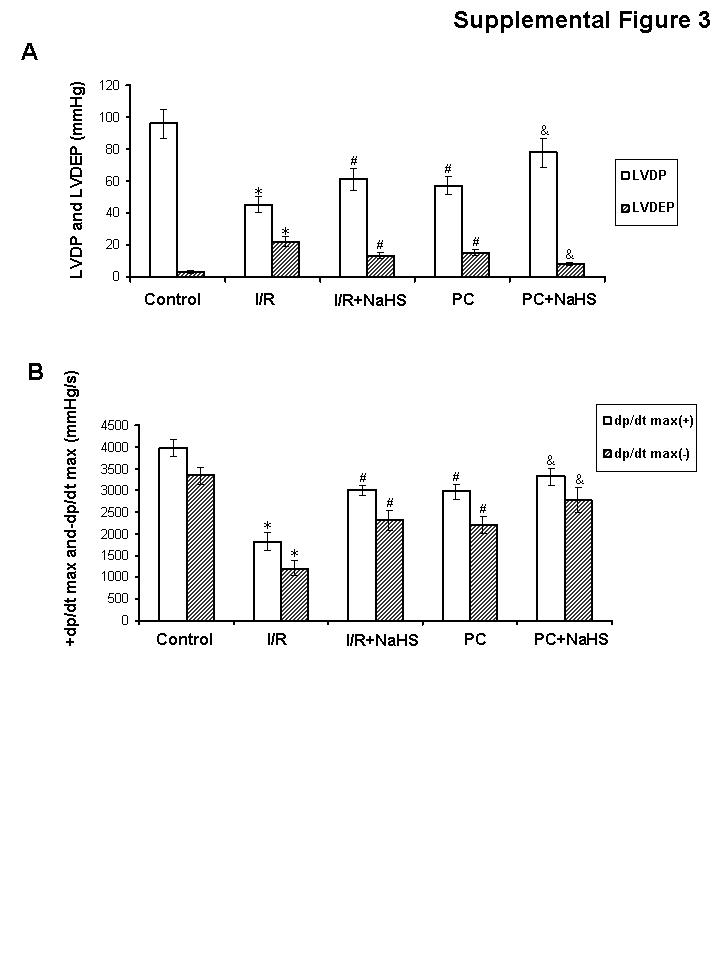


**Figure 3. The effect of exogenous H_2_S on cardiac function in the young rat hearts.**. LVDP, left ventricular developed pressure; LVEDP, left ventricular end-diastolic pressure; +dp/dt and -dp/dt, positive and negative maximum rate of left ventricular pressure development values are means ± S.E.M. of 8 determinations. * p<0.05 *vs*. control group; # p<0.05 *vs*. I/R group; & p<0.05 *vs*. PC group.


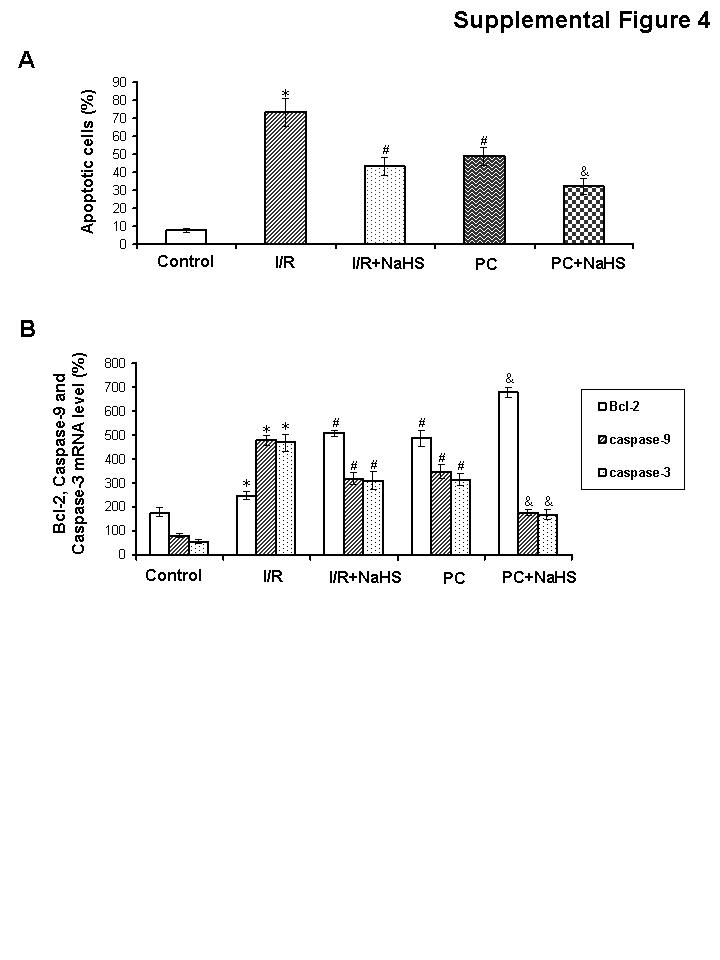


**Figure 4. The effect of exogenous H_2_S on apoptosis in the young rat hearts.** A. TUNEL staining detected cardiomyocytes apoptosis. B. The level of Bcl-2, caspase-9 and caspase-3 mRNA. The level of Bcl-2, caspase-9 and caspase-3 mRNA was tested using Real-Time PCR. The data were normalized to the GAPDH. All data were from four independent experiments. * p<0.05 *vs*. control group; # p<0.05 *vs*. I/R group; & p<0.05 *vs*. PC group.


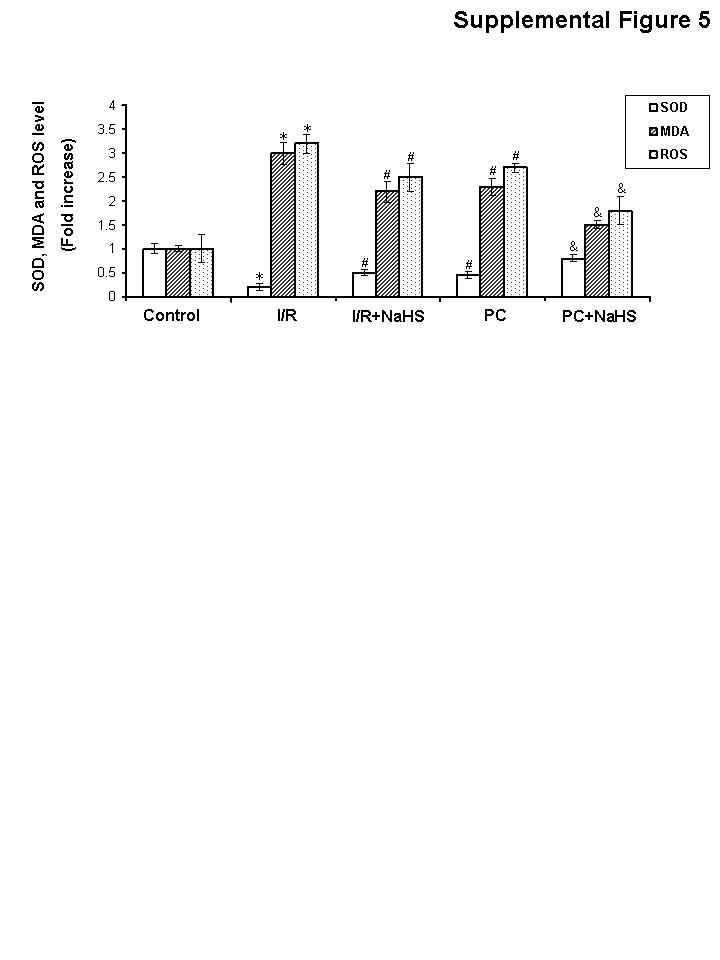


**Figure 5. The effect of exogenous H_2_S on the level of SOD, MDA and ROS in the young rat hearts.** All data were from eight independent experiments. * p<0.05 *vs*. control group; # p<0.05 *vs*. I/R group; & p<0.05 *vs*. PC group.
